# An international perspective on improving occupational conditions for direct care workers in home health

**DOI:** 10.1186/s13584-018-0249-5

**Published:** 2018-08-20

**Authors:** Miriam Ryvicker

**Affiliations:** Center for Home Care Policy and Research, Visiting Nurse Service of New York, 5 Penn Plaza, 12th Floor, New York, NY 10001 USA

**Keywords:** Home health care, Direct care workers, Home health aides, Occupational health and safety, Older adults

## Abstract

The occupational health and safety of direct care workers in the home health setting has been the focal point of a somewhat scarce, though highly important, body of research. Although the demand for home care services continues to expand with the rapidly growing population of older adults worldwide, home care workers - such as home health aides and personal care attendants - do not have the same level of protections by workplace safety policies such as those implemented in hospitals and nursing homes. This commentary synthesizes international perspectives on the occupational health and safety of home care workers, including the problem of workers’ rights violations and abuse by clients and their families. Prior policy and practice efforts have focused on improving the training, supervision, job satisfaction, and retention of home care workers, but have focused less on addressing issues of abuse. This paper recommends potential strategies to be developed and tested to provide a stronger support system for home care workers, more fully integrate them into the care team, and improve the occupational health and safety of this diverse, rapidly expanding workforce.

The Recent paper by Green and Ayalon [1] makes a valuable contribution to the evidence base in a critical area of concern for health policy – the wellbeing and rights of home care workers, including the particular vulnerabilities of migrant care workers. The study examines home care workers’ experiences of workers’ rights violations and abuse in the context of elder care in Israel. In this setting, older adults with functional impairments are eligible to receive government-funded home care services provided by either locally residing direct care workers on a part-time basis, or around-the-clock, live-in workers, depending on the severity of the person’s needs. A striking feature of this arrangement is that live-in home care is provided only by migrant workers, largely from the Philippines. Although the authors found that both groups of care workers – local, live-out and migrant, live-in – were frequently exposed to workers’ rights violations, migrant workers were more vulnerable to exploitation and emotional abuse by clients and their families. The conditions of live-in migrant workers are compounded by low Hebrew-language proficiency and the financial distress of debts accrued to pay brokers’ fees in the process of obtaining a work permit upon migrating to Israel.

The findings reported in this study reflect the particular intersection of long-term care payment and immigration policies as played out in the Israeli context under locally specific circumstances. Yet, the question of how to better protect and support the rapidly growing direct care workforce is global. In the United States, roughly 3 million direct care workers – commonly referred to as “paraprofessionals,” including certified home health aides and personal care attendants – provide care to older adults and individuals with disabilities in their homes [2]. According to the National Home Health Aide Survey conducted in the US in 2007, this workforce is predominantly female and low-income, with more than half receiving public assistance at some point in their lives [3]. Roughly half are racial/ethnic minorities [3], though this proportion is greater in large metropolitan areas [4]. As in Israel, immigrants play an important role in the home care workforce in both the US and Canada, especially in urban areas [4, 5].

The occupational health and safety of home care workers has been the focal point of a somewhat scarce, though highly important, body of research [3–19]. Home care workers do not have the same level of protections by workplace safety policies such as those implemented in hospitals and nursing homes [8]. A study of 1249 home care aides in the US found that 7% of the sample experienced physical violence on the job, while 19% were victims of verbal violence, including being yelled at, threatened, and subjected to racist language [10]. Another US study found that 20% of home care aides experienced some form of abuse on behalf of clients or clients’ families – including verbal abuse, prejudicial comments, or witnessing elder abuse or neglect. Moreover, those who experienced or witnessed abuse were more likely to report symptoms of depression in a 6-month follow-up interview [8]. In addition to the risk of verbal and emotional abuse, 19% of home care aides experienced at least one work-related injury in the past year, according to the National Home Health Aide Survey conducted in the US. These workers had lower job satisfaction and higher intent to leave the job [9]. It is worth noting that these US-based studies are focused mainly on home care aides employed by agencies certified by Medicare and Medicaid, the major government insurance programs in the US covering services provided to elderly and disabled individuals. There is also a substantial “gray market” of informal, unregulated direct care provided at lower rates to individuals in their homes, due to the limited insurance coverage for home-based long-term care [20]. As of yet, little evidence is available on the occupational conditions and potential abuses faced by workers in the gray market sector.

With the expanding demand for home care to address the needs of the aging population, it becomes increasingly important to find innovative ways to organize and regulate the provision of home care with an eye on quality of care, providing appropriate benefits and protections for home care workers, and cost efficiency. In the US, there has been growing interest in improving the quality of work life, training, job satisfaction and retention in the direct care workforce, given concerns about the negative impact of high turnover on quality of care [21–23]. However, addressing issues of worker abuse has not been a central focus of these efforts. In 2002–2006, the Better Jobs Better Care Demonstration (BJBC) was implemented across five states to improve job quality and retention for direct care workers in both home care and nursing homes [14]. The initiatives across participating states included training in communication skills, peer mentoring, supervisor coaching, career-ladder development, and other strategies. The evaluation of BJBC found mixed results overall, with little evidence of improved job satisfaction and retention [14, 24, 25]. More recently, New York State has been developing a new career ladder for an Advanced Home Health Aide role, to better serve the needs of patients with chronic conditions who have a high risk for potentially preventable, costly hospitalizations and poor health outcomes [26]. Moreover, beginning in 2018, New York has allocated state funding to the establishment of several Workforce Investment Organizations (WIOs), which involve partnerships among home care provider organizations, unions, managed care plans and other stakeholders [27]. The purpose of the WIOs is to better prepare the long-term care workforce to meet state objectives to improve patient outcomes in selected measures of high priority to managed care plans, which are undergoing significant transformation under health care payment reform. The WIO efforts include various initiatives to enhance the training of home health aides and create new potential career paths for them, such as the role of a health coach.

In our own evaluation of a home care workforce initiative implemented at three agencies in New York City (NYC), we found a significant increase in home health aide job retention, although there was still room for improvement in workers’ experiences [4]. Qualitative findings suggested that the quality of communication and the relationship with one’s supervisor was a significant concern for home health aides. However, the single most commonly reported area for improvement was in workers’ schedules and compensation. Unlike in Israel, where elderly clients frequently receive long hours or full-time aide services covered by government funding, home health aides in the US frequently work shorter, fragmented hours across multiple clients due to the structure of reimbursement by Medicare and Medicaid. Home health aides in the NYC context frequently reported that they were assigned too few hours to make ends meet, while having to pay for transportation between clients and no paid vacation time [4]. Although these conditions were likely exacerbated by the high cost of living in NYC, the fragmentation of work hours for home health aides is likely a national problem, given overall trends in Medicare and Medicaid reimbursement. Therefore, efforts to improve job quality for home health aides in the US will likely be limited without changes in the financial model of home health reimbursement to better align with the needs of the direct care workforce.

These prior efforts to better understand and improve the conditions of the direct care workforce offer several insights into the problem of abuse experienced by home care workers. First, the social and economic vulnerabilities of home care workers are a global issue. Whether due to immigration status, relying on public assistance, or being a racial/ethnic minority, home care workers frequently face societal barriers that likely make it even more difficult to address issues of abuse experienced in a patient’s home. Direct home care workers need to be provided with strategies for protecting themselves in harmful situations without risking a decrease in pay or job loss. Second, it is essential that home care organizations work to improve the relationships and communication between direct care workers and their supervisors. Although prior research has emphasized that the quality of one’s relationship with their supervisor is an important driver of job satisfaction [12, 14], it may also be key to addressing issues of worker abuse. Home care organizations could engage in training to raise awareness among supervisors of the risks of abuse faced by direct care workers and develop strategies to better support them.

Third, while many home care organizations deploy electronic health records for their clinical staff, home care aides – who spend the most hours with home care patients – are less likely to have access to an electronic record system for documenting interactions with the patient. Providing home care workers with technology to facilitate communication with supervisors in real time and to document encounters with patients could help to improve occupational safety and wellbeing, in conjunction with increased awareness and support from supervisors. Although these resources would be a substantial investment for the home care industry, the costs should be weighed against the cost of high turnover. Research is needed to establish a model for implementing, financing, and testing the adoption of an electronic system and supervision intervention to better integrate direct care workers into the rest of the home care team and address situations of abuse and other unsafe conditions in the patient’s home.

## Conclusions

The question of how to best care for the diverse, rapidly expanding workforce of home care aides has been persistent in recent decades, as the need for high quality care in the home continues to expand with the growing population of older adults and individuals with disabilities [7, 14]. Advancements in communication technology offer a potential opportunity to better connect home care workers to a support system. Establishing such a support system would call for both structural and cultural shifts in the home care industry to raise awareness and provide practical solutions to improve the occupational health and safety of home care workers both locally and globally.

## Abbreviations

BJBC: Better jobs better care
NYC: New York City
US: United States
WIO: Workforce investment organization
